# Public attitudes in Japan toward participation in whole genome sequencing studies

**DOI:** 10.1186/s40246-018-0153-7

**Published:** 2018-04-13

**Authors:** Taketoshi Okita, Noriko Ohashi, Daijiro Kabata, Ayumi Shintani, Kazuto Kato

**Affiliations:** 1 Graduate School of Medicine, Tohoku University, 2-1 Seiryo-machi, Aoba-ku, Sendai, 980-8575 Japan; 2 Graduate School of Medicine, Osaka University, 2-2 Yamadaoka, Suita, Osaka 565-0871 Japan; 3 Graduate School of Medicine, Osaka City University, 1-4-3, Asahi, Abeno, Osaka, Osaka 545-8585 Japan

**Keywords:** Whole genome sequencing studies, Public attitudes, Japan, Participation in WGS studies, Gene-related information

## Abstract

**Background:**

Recent innovations in gene analysis technology have allowed for rapid and inexpensive sequencing of entire genomes. Thus, both conducting a study using whole genome sequencing (WGS) in a large population and the clinical application of research findings from such studies are currently feasible. However, to promote WGS studies, understanding and voluntary participation by the general public is needed. Therefore, it is essential to investigate the general public’s attitude toward and understanding of WGS studies. The primary goal of our research is to investigate these issues and to discover how they relate to research participation in WGS studies.

**Methods:**

A survey of awareness regarding WGS and studies using WGS was conducted with a sample of 2000 or more participants using a self-administered questionnaire posted on the Internet between February 20 and 21, 2015. Prior to the survey, we briefly explained WGS and WGS study-related issues to the respondents in order to provide them with the minimum knowledge required to answer the questionnaire. We then conducted an analysis, including cross-classification.

**Results:**

For the question regarding interest in WGS, 46.6% of participants responded “Yes.” 70.7% of all respondents said that they were interested in some kinds of findings that could be obtained from WGS studies. Regarding participation in WGS studies, 29.0% were interested in participating. The demographic factors significantly related to attitudes toward research participation were age, level of education, and employment status. The results also suggest that concerns about WGS have a positive effect on people’s willingness to participate. Furthermore, it was shown that for people who were not interested in their gene-related information, concerns about WGS negatively impacted their willingness to participate. However, for people who were interested in their gene-related information, their concerns might not have impacted their willingness to participate.

**Discussion and conclusions:**

This research has shown several key factors that affect the willingness of the general public for the participation to the WGS studies. One of the unexpected findings is that concerns toward WGS studies generally have positive effect on the peoples’ attitude. It will be interesting to further investigate into the various types of concerns that people in different groups have about WGS.

## Background

Significant advances in genome analysis have led to increased understanding of diseases related to genome mutation [1]. Such advances have also allowed researchers to obtain a variety of gene-related information, including information associated with drug responsiveness, physical constitution, and blood relationships [2, 3]. In particular, recent innovations in gene analysis technology have allowed for rapid and inexpensive sequencing of entire genomes [4]. Thanks to these developments, conducting studies using whole genome sequencing (WGS) in a large population and applying these research findings in clinical settings have become possible. However, to promote WGS studies and use their findings more widely in clinical settings, increased participation from the general public in WGS studies is needed. In addition, the need for domestic and international research collaboration and data sharing across projects and institutions has been rapidly growing [5].

In response to this trend, new issues associated with WGS studies have emerged, including the sharing of genome information obtained through WGS (data sharing) and returning personal genetic information to subjects (return of results).

In order to conduct future investigations using WGS in a large study population in Japan, and to make effective regulatory policies, we believe that knowledge regarding attitudes toward and understanding of WGS and studies using WGS needs to be obtained. Several reports on WGS awareness surveys conducted in Japan and in other countries have been published [6, 7], and public attitudes on WGS data sharing [8–15] and return of such results [16–19] have been investigated. However, public attitudes toward participation in WGS studies have never been reported in Japan.

In this study, after the respondents had received a summary explanation of WGS, we conducted a survey of people’s interest and concerns regarding WGS, their willingness to participate in WGS studies, and background factors correlated with their attitudes, in order to aid in the design of future WGS studies.

## Methods

### Study design

A survey of awareness around WGS and studies using WGS was conducted using a self-administered questionnaire posted on the Internet. Prior to each section where respondents choose their answers, we briefly explained WGS and WGS study-related issues, including an outline of WGS studies, data sharing, and return of results, to the respondents to provide them with the minimum knowledge required to answer the questionnaire.

The survey was conducted with a target number of 2000 or more participants, in accordance with the demographic distribution shown in the Japanese national census between February 20 and 21, 2015. Video Research Ltd. was in charge of the visual layout of the questionnaire and the implementation of the survey.

### Recruitment participants

Participants were recruited from the general public who were registered as part-time survey assistants with Video Research Ltd., to which we outsourced part of the survey.

The subjects comprised males and females aged 16 years or older with a wide variety of attributes, but who matched with the age distribution of the Japanese population based on the Japanese government’s national population census in 2010. Only the responses from the subjects who completed the entire questionnaire were included, and enrollment was considered to have been completed when the target number of the respondents was reached.

We received all the results of the survey from Video Research Ltd. and conducted an analysis, including cross-classification.

### Statistical analysis

All data were summarized using medians and interquartile ranges for continuous variables whereas counts and percentages for categorical variables.

To identify factors associated with the willingness to participate in WGS studies, a proportional-odds logistic regression model was used with inclusion of age, sex, income, family number, education, employment, and marriage as explanatory variables. Non-linear restricted cubic splines were used to assess non-linearity of all continuous variables. Missing data were imputed using the multiple imputation methods using areg.impute function in rms package of R software [20].

In order to examine the effect of each variable indicating subjects with or without interest in and concerns regarding WGS, as well as those who did or did not wish to get the gene-related information on willingness to participate in WGS studies, similar analyses were performed.

To discover whether the effect of concerns about WGS was influenced by whether or not a subject wanted to get gene-related information, the interaction between these two variables was assessed with inclusion of their cross-product terms in the multivariable regression along with the main effect variables.

All statistical inferences were made two-sided at the 5% significance level. All statistical analyses were performed with the R software (version 3.2.2, R Foundation for Statistical Computing, Vienna, Austria) using the rms package.

### IRB approval

Institutional review board (IRB) approval was obtained at the Osaka University and Tohoku University prior to implementation of this study.

## Results

### Demographic characteristics (socio-demographic)

The demographic characteristics of the participants are presented in Table 1. A total of 2399 participants were included in the analysis.

**Table 1:** Participant characteristics

Characteristic	All participants (*N* = 2399)
Age	45.00 (33.00, 57.00)
Gender	
Male	1196 (49.9%)
Female	1203 (50.1%)
Education	
Junior high school	71 (3.0%)
High school	711 (29.6%)
Professional school	267 (11.1%)
Technical college	24 (1.0%)
Junior college	250 (10.4%)
University	952 (39.7%)
Graduate school	114 (4.8%)
Other	10 (0.4%)
Employment status	
Permanent employment	864 (36.0%)
Part-time or temporary employment	416 (17.3%)
Student	165 (6.9%)
Full-time homeworker	454 (18.9%)
Retired	80 (3.3%)
Unemployed	251 (10.5%)
Other	169 (7.0%)
Annual income, JPY	
< 2,000,000	277 (14.0%)
2,000,000–3,000,000	252 (12.8%)
3,000,000–4,000,000	278 (14.1%)
4,000,000–5,000,000	278 (14.1%)
5,000,000–6,000,000	213 (10.8%)
6,000,000–7,000,000	193 (9.8%)
7,000,000–8,000,000	127 (6.4%)
8,000,000–10,000,000	165 (8.4%)
≧ 10,000,000	192 (9.7%)
Number of people in household	3.00 (2.00, 4.00)
Marital status	
Unmarried	926 (38.6%)
Married	1309 (54.6%)
Divorced or widowed	164 (6.8%)

The population comprises 1196 males (49.9%) and 1203 females (50.1%), with a mean age of 44.91 years. While it is difficult to make direct comparisons with the national census data and the results from our study, concerning educational background, we note the following differences: while those who studied until high school make up for 46.5% of people in the 2010 national census [21], in our study, only 29.6% of respondents ended their education with high school. Furthermore, while university and graduate school graduates only make up 19.9% of people in the census data, 44.5% of our respondents had graduated from either university or graduate school. It is clear from the above that the respondents in our survey had comparatively higher educational backgrounds than the general population. Concerning employment, the 2015 national census showed that approximately 16.7% (20 million) of people had part-time or temporary employment, while approximately 27.5% (33 million) had permanent employment [22]. In our survey, 17.3% of respondents were non-regularly employed and 36.0% had regular employment. Compared to the general population, more of the respondents had permanent employment. However, unemployment rates were higher among respondents in our survey (10.5%) than in the general population (3.4%), according to the 2015 national census.

### Interest in and concerns about WGS

After we provided a brief explanation of WGS, the participants answered the questions, including questions regarding their interest in and concerns about WGS, and what type of information they would have interest in if their own sample was to be analyzed using WGS (see Tables 2 and 3). However, these questions were not limited to participants’ interest or concern about studies specifically (such as returning incidental findings), but rather attempted to clarify their attitude toward or opinion of WGS in general.

**Table 2:** Interest in and concerns about whole genome sequencing

	All participants (*N* = 2399)
Interest	
Very much	277 (11.5%)
Moderately	841 (35.1%)
Neutral	625 (26.1%)
A little	275 (11.5%)
Not at all	381 (15.9%)
Concern	
Very much	119 (5.0%)
Moderately	573 (23.9%)
Neutral	947 (39.5%)
A little	403 (16.8%)
Not at all	357 (14.9%)

**Table 3:** Information participants wish to know

	All participants (*N* = 2399)
Disease-related information	1342 (55.9%)
Information on gene-related drug effectiveness and side effects	698 (29.1%)
Information on physical constitution, such as types prone to obesity or inebriety	1047 (43.6%)
Information on aptitudes and abilities	631 (26.3%)
Information on family relations	171 (7.1%)
There is no gene-related information I wish to know	702 (29.3%)

For the question about interest in WGS, the participants responded as follows: 46.6% (*n* = 1118) “Yes (Very much or Moderate),” 26.1% (*n* = 625) “Neutral,” and 27.4% (*n* = 656) “No (A little or Not at all).” For the question of whether they have concerns regarding the establishment of WGS technology: 28.9% (*n* = 692) chose “Yes (Very much or Moderate),” 39.5% (*n* = 947) chose “Neutral,” and 31.7% (*n* = 760) chose “No (A little or Not at all).”

For the question, “what type of information would you wish to know if your own sample was analyzed using WGS” (multiple answers), the most frequent answer was “disease-related information (55.9%, *n* = 1342),” the second was “information on physical constitution, such as types prone to obesity and inebriety (43.6%, *n* = 1047),” the third most frequent was “information on gene-related drug effectiveness and side effects (29.1%, *n* = 698),” the fourth was “information on aptitudes and abilities (26.3%, *n* = 631),” and the fifth was “information on family relations (7.1%, *n* = 171).” Overall, the top 3 were information related to physical conditions. Notably, the answer “there is no gene-related information I wish to know” accounted for a relatively high percentage of answers (29.3%, *n* = 702).

### Willingness to participate in WGS studies

The participants were asked to answer questions regarding their willingness to participate in WGS studies after they were provided with a brief introduction to WGS-related issues, including data sharing and return of results. This survey revealed that 29.0% of the participants (*n* = 696) were willing (“Yes—Very much or Moderate),” 33.6% (*n* = 806) were “Neutral,” and 37.4% (*n* = 897) were unwilling (“No—A little or Not at all)” (see Table 4). Regarding willingness to participate in WGS studies, more respondents were unwilling to participate than those willing to participate.

**Table 4:** Willingness to participate in the whole genome sequencing study

	All participants (*N* = 2399)
Very much/Moderately	696 (29.0%)
Neutral	806 (33.6%)
A little/Not at all	897 (37.4%)

### Background factors correlated with willingness to participate in WGS studies: demographic factors

The demographic factors of age, level of education, and employment status were found to be significantly correlated with willingness to participate in WGS studies. However, we were unable to find any significant correlation with sex, income, number of family members, or marriage.

**Fig. 1:**
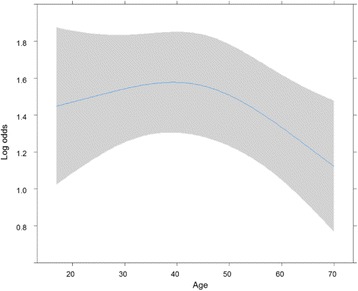
The effects of age on willingness

For age (see Fig. 1), willingness to participate in WGS studies decreased with increasing age (*p* < 0.05). However, as shown in Fig. 1, although the *p* value was marginal (*p* = 0.062), the curve based on logistic analysis revealed that the people around 40 years of age showed the most positive attitude toward participation in WGS studies, but this attitude became increasingly negative with age after this point.

Higher levels of education were associated with positive attitudes toward participation in WGS studies for some comparisons. More specifically, compared with university graduates, significant differences were observed for graduates from junior high schools (*p* < 0.001), high schools (*p* < 0.001), and professional schools (*p* < 0.05), but no difference was found for the graduates from technical colleges, junior colleges (which, in Japan, are considered as comparable to universities), and graduate schools. These results suggested that education levels, especially university graduate level or higher, could affect attitudes toward participation in WGS studies.

For employment status, compared with permanent employment as a reference, only the student group significantly showed a positive attitude (*p* < 0.05).

### Background factors correlated with willingness to participate in WGS studies: attitudes toward WGS

For the relationship between the general interest in WGS and attitudes toward participation in WGS studies, the *p* value was significant (*p* < 0.001). The people who had concerns about WGS were also more willing to participate in WGS studies (*p* < 0.001).

For the relationship between interest in the information available from WGS and attitudes toward participation in WGS studies, distinct differences were observed between the people who wished to know gene-related information and the people who did not wish to know any gene-related information. The respondents who answered that they were interested in knowing their gene-related information (70.7%, *n* = 1697) showed more positive attitudes toward participation in WGS studies than those who did not have interest in such information (29.3%, *n* = 702) (odds rate = 10.3, *p* < 0.001).

Furthermore, analysis of the effect of concerns about WGS on the attitudes toward participation in WGS studies revealed that in the group of people who were interested in their gene-related information, any such concerns were unlikely to significantly influence willingness to participate in WGS studies (*p* = 0.943). In contrast, in the group of people not interested in any information from WGS, willingness to participate in WGS studies was significantly lower in the people with concerns about WGS than in those with no such concerns (*p* = 0.021).

## Discussion

In this awareness survey conducted among members of the Japanese general public, almost half of the participants answered that they had an interest in WGS. The level of their interest was not necessarily low. However, many also showed negative attitudes toward participation in WGS studies. Our survey has showed that in Japan, there might exist a wide gap between people’s interest in WGS and their willingness to participate in WGS studies.

However, since we can observe significantly positive attitude toward participation in WGS studies among students and younger people, if their attitudes toward WGS continue as they are, it is possible that the number of active participants in WGS research will increase in the future. Incidentally, the willingness to participate in WGS research was the strongest among people in their 40s. However, this trend showed a decrease past this point. One possible explanation for this is that people are getting married later and later in Japan, and thus, many people in their 40s are most concerned about the responsibility for their families and society. This fact may have some positive impact on their willingness to participate in WGS studies.

Another study performed in Japan (Ishiyama et al.) showed that men had significantly more positive attitudes toward genomic studies than women [6]. However, in our research, we were unable to observe this trend. Since both our method and subject differ from those of Ishiyama et al., it is difficult to make a simple comparison of our results. One possibility is that sex is becoming a weaker factor in terms of attitudes toward genomic and other kinds of scientific research.

The main factor correlated to a respondent’s willingness to participate in WGS studies was whether or not they had interest in gene-related information. The group which expressed interest in gene-related information—compared to the group which did not—was much more likely to have a positive attitude toward participation in WGS research.

Additionally, the results suggest that for the group of people who are not interested in their gene-related information, concerns about WGS negatively impact their willingness to participate in WGS studies. However, if people are interested in knowing their gene-related information, their concerns might not impact their willingness to participate in WGS studies, or these concerns might even positively impact their interest in the information to be gained from WGS and their willingness to participate in WGS studies. Therefore, we can safely say that having an interest in gene-related information functions as a very strong and stable factor in whether or not a person is willing to participate in WGS research.

Our findings regarding the effect of people’s concerns toward WGS were also of interest. Generally speaking, concerns about WGS would be expected to have a negative effect overall on people’s willingness to participate. However, in our study, these concerns exert different effects depending on whether the person is interested in gene-related information or not. For those who are interested in gene-related information, these concerns do not affect their willingness to participate—in fact, they may positively influence the person’s willingness. For those who are not interested in gene-related information, concerns about WGS seem to exert a negative influence on their willingness to participate. Considering the complexity of these results, it is necessary to understand and analyze the specific kinds of concerns that people have about WGS and whether these concerns are the same among those interested in gene-related information and those who are not.

### Limitations

The level of education of the respondents in this study was higher than that shown in the national population census. This might be because the survey was conducted using a self-administered questionnaire posted on the web and because a scientific research topic, WGS, was the subject of the survey. In addition, the unemployment rate referenced in this survey (10.5%) seems much higher than Japan’s actual rate of unemployment (3.4% at the time of writing). We suspect that this number may include some part-time workers. However, we are unable to investigate this statistic further within the scope of this paper.

## Conclusion

The results suggest, based on the correlation analysis between the background factors and willingness to participate in WGS studies, that if a person has interest in knowing their gene-related information, this could be a major factor in improving their willingness to participate in WGS studies. Additionally, concerns about WGS did not always negatively impact the willingness to participate in WGS studies and thus could be a potential factor in increasing willingness. For these reasons, it is essential that we continue our investigation into the kinds of concerns that people have about WGS, as well as whether these concerns are shared between people who are interested in gene-related information and those who are not.
